# Adhesive-Free Adhesion between Plasma-Treated Glass-Cloth-Containing Polytetrafluoroethylene (GC–PTFE) and Stainless Steel: Comparison between GC–PTFE and Pure PTFE

**DOI:** 10.3390/polym14030394

**Published:** 2022-01-20

**Authors:** Misa Nishino, Yuki Okazaki, Yosuke Seto, Tsuyoshi Uehara, Katsuyoshi Endo, Kazuya Yamamura, Yuji Ohkubo

**Affiliations:** 1 Graduate School of Engineering, Osaka University, 2-1 Yamadaoka, Suita, Osaka 565-0871, Japan; m-nishino@div1.upst.eng.osaka-u.ac.jp (M.N.); y-okazaki@div1.upst.eng.osaka-u.ac.jp (Y.O.); y-seto@div1.upst.eng.osaka-u.ac.jp (Y.S.); endo@upst.eng.osaka-u.ac.jp (K.E.); yamamura@prec.eng.osaka-u.ac.jp (K.Y.); 2 SEKISUI CHEMICAL Co., Ltd., 2-2 Kamichoshi-cho, Kamitoba, Minami-ku, Kyoto 601-8105, Japan; Uehara_Tsuyoshi@sekisui.com

**Keywords:** adhesion, fluoropolymers, metals, plasma, glass-cloth-containing polytetrafluoroethylene

## Abstract

In this study, the effect of plasma treatment on glass-cloth-containing polytetrafluoroethylene (GC–PTFE) was investigated. Previous plasma studies investigated pure PTFE (which does not contain glass cloth) but not GC–PTFE. The effect of Ar + H_2_O plasma treatment on GC–PTFE was investigated. The Ar + H_2_O plasma-treated GC–PTFE sheets were thermally compressed to stainless steel (SUS304) foils without using adhesive, and the GC–PTFE/SUS304 adhesion strengths were measured using a 90° peel test. The adhesion strength increased with the increase in the plasma treatment time (0.8 and 1.0 N/mm at 20 s and 300 s, respectively). Thus, strong adhesion between GC–PTFE/SUS304 was achieved without adhesive. This improvement in the adhesion properties of GC–PTFE can be attributed to the generation of oxygen-containing functional groups and the decrease in the surface roughness of the samples. Thereafter, the adhesion properties of GC–PTFE and pure PTFE were compared. Because, unlike pure PTFE, GC–PTFE has no weak boundary layer, GC–PTFE exhibited better adhesion properties than pure PTFE under short plasma treatment times.

## 1. Introduction

Polytetrafluoroethylene (PTFE) has several excellent properties, such as high hydrophobicity, lipophobicity, chemical resistance, and tribological properties [1]. Therefore, PTFE is used to fabricate the sliding parts of printers and sliding bearing materials for base isolation. Pure PTFE sheets with no reinforcements, e.g., carbon fibers, glass fibers, and glass cloth (GC), are manufactured by compressing the PTFE powder and then cutting the bulk PTFE material into sheets [2]. A weak boundary layer (WBL) [3,4] is introduced on the surface of the PTFE at the time of cutting. A WBL on the surface of the PTFE promotes the peeling of other materials from the PTFE surface as WBL reduces the adhesion strength regardless of the presence or absence of surface treatment. In addition, PTFE is a material with low surface free energy [5,6,7]. Therefore, WBL removal and the generation of oxygen-containing functional groups are necessary for PTFE adhesion onto other materials. GC-containing PTFE (GC–PTFE) is prepared by immersing a GC in a dispersed solution of PTFE and then baking it. Thus, cutting is not required for manufacturing GC–PTFE sheets, and WBLs are not introduced on the GC–PTFE surface. Consequently, a decrease in the surface treatment time is expected when pure PTFE is replaced with GC–PTFE because the WBL removal step is not conducted in this process. However, the surface treatment of GC–PTFE has not been investigated in detail.

Wet and dry surface treatments of pure PTFE have been previously reported. The wet process involves chemical etching using a solution containing sodium (Na). This process drastically improves the adhesion properties of PTFE in a short treatment time because of the strong reduction power of metallic Na [8]. However, chemical etching has several disadvantages, such as a high environmental impact and PTFE discoloration. The dry process can be performed using several methods, involving either ion radiation, ultraviolet (UV), electron beam (EB), or plasma. Ion radiation using Ar^+^ ion at 1 keV was reported to improve the adhesion properties of pure PTFE to Cu. However, the precise adhesion strength was not reported [9]. Furthermore, it was reported that oxygen-containing functional groups were generated on a pure PTFE surface by low-energy nitrogen-ion irradiation at 300 eV, and the adhesion strength between pure PTFE and a GC tape using an epoxy adhesive increased from 0.02 to 0.9 N/mm [10,11]. However, to the best of our knowledge, no studies have reported a strong adhesion of ion-irradiated pure PTFE or GC–PTFE onto different materials with no adhesive. Surface treatment using UV irradiation was reported to increase the oxygen-containing functional groups on the pure PTFE surface. Moreover, the surface free energy of pure PTFE was increased by UV irradiation in an oxygen atmosphere. However, the precise adhesion strength was not reported [12]. In addition, it was reported that the adhesion strength between pure PTFE and polymethylmethacrylate (PMMA) using an epoxy adhesive increased from 0.03 to 10 MPa by coating the UV-irradiated pure PTFE with 1,2-diaminoethane [13], and the adhesion strength between pure PTFE and plexiglass using an epoxy adhesive increased from 0.03 to 10 MPa by coating the UV-irradiated pure PTFE with triethylenetetramine [14]. Treating pure PTFE with a combination of UV irradiation and coating improves its adhesion properties; however, these results are limited to using coating reagents and adhesives. To the best of our knowledge, there are no reports on the strong adhesion of UV-irradiated pure PTFE and different materials without an adhesive. EB irradiation increased the oxygen-containing functional groups and C–C crosslinks generated on the surface of pure PTFE [15,16,17]. In addition, sulfone groups can be introduced onto the pure PTFE surface through a grafting reaction with styrene monomer, acrylic acid, or sodium 4-styrene sulfonate after EB irradiation [18,19]. However, EB irradiation has been reported to cause a chain scission of the CF_2_ chains in pure PTFE, decreasing the crystallinity and crystallite size [15,20], and increasing the surface roughness [16,21]. Moreover, outermost surface modification of pure PTFE using EB irradiation is difficult because the penetration depth of EB is 100–150 μm [22]. The adhesion strengths between EB-irradiated pure PTFE and carbon fiber reinforced plastics (CFRP) and between EB-irradiated pure PTFE and polydimethylsiloxane (PDMS) were both approximately 0.1 N/mm [22,23]. No studies have reported strong adhesion between an EB-irradiated pure PTFE and different materials. Plasma treatments using Ar + O_2_, N_2_ + H_2_, He + H_2_O, Ar + H_2_O, and Ar + NH_3_-H_2_O as the process gas were conducted with pure PTFE [24,25,26,27,28,29,30,31]. Several studies reported an indirect adhesion between plasma-treated pure PTFE and different materials using an adhesive. For example, the adhesion strength between pure PTFE and an iron sheet using an epoxy adhesive increased from approximately 0.3 to 5.8 MPa after an Ar + H_2_O plasma treatment for 120 s under low pressure (approximately 50 Pa) [29]. In addition, the maximum adhesion strength between pure PTFE and nitrile butadiene rubber using a phenolic adhesive and after a plasma treatment using NH_3_ for 10 min under low pressure (26.6 Pa) was 8.1 N/mm, which is higher than that of Na-treated pure PTFE (6.1 N/mm) [30]. The adhesion strength between pure PTFE and Al plate using an epoxy adhesive increased from 0.02 to 1.4 N/mm via Ar + acrylic acid plasma treatment under atmospheric pressure [31]. In addition, the adhesion strength between pure PTFE and stainless steel bar using an epoxy adhesive increased from 0.00 to 1.17 N/mm via heat-assisted Ar plasma under atmospheric pressure [32]. The direct adhesion between pure PTFE and different materials without any adhesives was also reported. The adhesion strength between pure PTFE and Cu was reported to increase from 0.03 to 0.48 N/mm after plasma polymerization using glycidyl methacrylate (GMA) [33]. In addition, an adhesion strength of 1.9 N/mm between pure PTFE and Cu was obtained by two-step graft polymerization using 1-vinylimidazole (VIDz) and GMA after Ar plasma treatment and followed by O_2_ plasma treatment [34]. Although these cases did not involve an adhesive, graft polymerization was required. The direct adhesion of pure PTFE to different materials, such as rubber and metals, with no graft polymerization or adhesives was also reported. For example, the adhesion strength between isobutylene–isoprene rubber (IIR) and pure PTFE treated with heat-assisted He plasma under atmospheric pressure increased from approximately 0.0 to > 2.0 N/mm, and cohesion failure of the rubber occurred during the peel test [35]. Moreover, the adhesion strength between Ag ink and pure PTFE increased from 0.04 to > 1.0 N/mm after open-air type Ar + H_2_O plasma treatment [36]. However, in both cases, the studies were focused on the plasma treatment of pure PTFE. To the best of our knowledge, there are no studies on the plasma treatment of GC–PTFE. In addition, the plasma treatment of pure PTFE with WBLs is generally time consuming [36]. In this study, the surface state of plasma-treated GC–PTFE is investigated. In addition, plasma-treated GC–PTFE and pure PTFE are compared to investigate the length of the plasma treatment time in the case of GC–PTFE, which has no WBL. The surface-modification conditions and adhesion properties for different cases of PTFE are listed in Table 1.

## 2. Materials and Methods

### 2.1. Materials

GC–PTFE sheets (No. 9700UL, Nitto Denko, Kita-ku, Osaka, Japan, thickness = 0.23 mm) and pure PTFE sheets (No. 900UL, Nitto Denko, Kita-ku, Osaka, Japan, thickness = 0.2 mm) were cut into 140 × 200-mm specimens and used as the PTFE samples. The glass fiber diameter inside the GC–PTFE was about 100 μm. Stainless steel SUS304 foils (3-2158-09, AS ONE, Nishi-ku, Osaka, Japan, thickness = 0.5 mm) were cut into 50 × 60 mm specimens and used as adherends to the GC–PTFE or pure PTFE. All PTFE and SUS304 samples were washed with acetone (99.5%, Kishida Chemical, Chuo-ku, Osaka, Japan) and pure water using an ultrasonic cleaner (US-4R, AS ONE, Nishi-ku, Osaka, Japan) for 1 min. After ultrasonic cleaning, all PTFE and SUS304 samples were dried using an N_2_ gun (99.99%, Iwatani Fine Gas, Amagasaki, Hyogo, Japan).

### 2.2. Plasma Treatment

GC–PTFE and pure PTFE sheets were plasma-treated using an open-air type plasma treatment system (AP-T05-L150, Sekisui Chemical, Minami-ku, Kyoto, Japan) with a direct current (DC)-pulse power supply and two electrodes (stainless steel) covered with an insulator (alumina) to obtain stable glow discharge. The output voltage, the gap between the electrodes and the surface of the PTFE samples, and the plasma treatment time were 10.7 kV, 0.7 mm, and 20–300 s, respectively. Ar (99.99%, Iwatani Fine Gas, Amagasaki, Hyogo, Japan) was used as the main process gas at a total flow rate of 15.0 L/min. Water vapor was used as the added gas at a flow rate of 15 mg/min. The Ar flow rate was controlled using a float flow meter (RK1250, KOFLOC, Kyotanabe, Kyoto, Japan). A vaporizer (MI-1141-PV, HORIBA, Minami-ku, Kyoto, Japan) was used to generate water vapor, and the mass flow rate of the added water vapor was maintained at 15 mg/min by feedback control using a mass flow controller (LF-F20M-A, HORIBA, Minami-ku, Kyoto, Japan). To stabilize the concentration of water vapor, it is important to deliver the water vapor to the plasma treatment system without it transforming into a liquid. To accomplish this, the gas pipes were heated at 110 °C using a ribbon heater (RB100-200-20-2000, THREE HIGH, Yokohama, Kanagawa, Japan), a digital temperature controller (monoone-120, THREE HIGH, Yokohama, Kanagawa, Japan), and a temperature sensor (TH-8297-1, THREE HIGH, Yokohama, Kanagawa, Japan). To prevent the temperature of the pipes decreasing, a silicone sponge square string (Sh3-30-3, THREE HIGH, Yokohama, Kanagawa, Japan) was wrapped around the metal pipes as a thermal insulator. The water vapor concentrate was measured using a dew-point meter (TK-100, TEKHNE, Kawasaki, Kanagawa, Japan). The saturated steam pressure was calculated using Equation (1), which is called Tetens’ formula [37].

E = 6.11 × 107.5 t/(237.3 + t)(1)

where t is the dew point and E is the saturated steam pressure.

The moisture content is given by Equation (2).

M = E/E’ × 100(2)

where M is the moisture content, E is the saturated vapor pressure, and E’ is the environmental pressure.

The remaining organic contaminants on the surface of the washed SUS304 foils were removed using a plasma jet (PJ) treatment device (Tough Plasma FPE-20, FUJI CORPORATION, Chiryu, Aichi, Japan). The gap between the PJ-emitting openings and the surface of the SUS304 foils was 20 mm. N_2_ was used as the main process gas at a flow rate of 29.7 L/min. An artificial air gas (Iwatani Fine Gas, Amagasaki, Hyogo, Japan) was used as the added gas at a flow rate of 0.3 L/min. The plasma irradiation area was approximately 20 mm × 10 mm when the sample stage was not scanned. This was not sufficient for the adhesion tests. Therefore, the plasma irradiation area was expanded to 20 mm × 60 mm by scanning the sample stage. A total of 10 scans were conducted at a scanning distance of 60 mm and a stage scanning speed of 0.8 mm/s.

### 2.3. Water Contact Angle (WCA) Measurements

The automatic contact angle meter (DropMaster300, Kyowa Interface Science, Niiza, Saitama, Japan) was used to investigate the WCA of the surfaces of PTFE samples before and after plasma treatment. The WCA was measured using pure water. A 3.0 μL droplet was used. After 1 s, the WCA was measured. The WCA measurements were repeated five times under each plasma treatment condition. The average of the five values was defined as the WCA, and the error bar shows the standard error.

### 2.4. X-ray Photoelectron Spectroscopy (XPS) Analysis

XPS (Quantum2000, ULVAC-PHI, Chigasaki, Kanagawa, Japan) was used to investigate the change in the chemical-bonding state of the surfaces of PTFE and SUS304 samples before and after plasma treatment. The cumulative number, pass energy, and step size were 3, 25.00 eV, and 0.05 eV, respectively. XPS measurements were conducted at an X-ray take-off angle of 45°. Both low-speed EB and low-speed Ar ion irradiation were used to avoid surface charging during XPS measurements. The measured XPS data were analyzed using data analysis software (MultiPak V8.2C, 2007-9-04). The peaks were indexed to CF_2_ at 292.5 [12] and 291.8 eV for the as-received PTFE and plasma-treated PTFE samples, respectively [38,39], and to C–C (C–H) at 284.6 eV for the SUS304 samples [38].

### 2.5. Confocal Laser Scanning Microscope (CLSM) Test

A CLSM (LEXT OLS4100, Olympus, Shinzyuku-ku, Tokyo, Japan) was used to investigate the surface roughness of the GC–PTFE before and after plasma treatment. The root mean square, Sq, and the arithmetic mean estimation, Sa, of the roughness were calculated for an area of 2567 μm × 2567 μm. Surface roughness measurements were repeated three times using the same samples, and the average of the three value was defined as the surface roughness. The Olympus Stream software ver. 1.1.7.2 was used.

### 2.6. Scanning Electron Microscope (SEM) Analysis

A field emission scanning electron microscope (FE-SEM, S4800, Hitachi High-Tech, Minato-ku, Tokyo, Japan) was used to compare the surface morphologies of GC–PTFE and pure PTFE samples before and after plasma treatment at an acceleration voltage of 5 kV. The surfaces of PTFE samples were coated with a thin Au film using a sputtering apparatus (Smart Coater, JEOL, Akishima, Tokyo, Japan) to avoid surface charging. The Au deposition rate was 5 nm/min when the gap between the samples and target was 20 mm. The thickness of the thin Au film was estimated at 10 nm because the sputtering time was 2 min.

### 2.7. Adhesion Strength Measurements Using a 90° Peel Test

PTFE samples were directly adhered to SUS304 samples using thermal compression without any adhesives to measure the adhesion strengths between the PTFE and SUS304 samples. The plasma-treated PTFE samples were sandwiched between the untreated and PJ-treated SUS340 foils. This was then sandwiched between two plates and compressed under 2 MPa at 320 °C for 10 min using a compression molding machine (H400-15, AS ONE, Nishi-ku, Osaka, Japan). The adhesion strength between PTFE and SUS304 samples was measured using a 90° peel test. Samples were fixed using an electrically driven test stand (MX-500N, IMADA, Toyohashi, Aichi, Japan), and adhesion strength measurements were conducted using a digital force gauge (ZP-200N, IMADA, Toyohashi, Aichi, Japan). The plasma treatment time was varied between 20 and 300 s for PTFE samples, and the PTFE/SUS304 adhesion strength measurements were repeated three times under each plasma treatment condition. The average of the three values was defined as the adhesion strength, and the error bar shows the standard error.

## 3. Results and Discussion

### 3.1. Assessment of the Wettability of GC–PTFE Using the WCA Measurement

**Figure 1:**
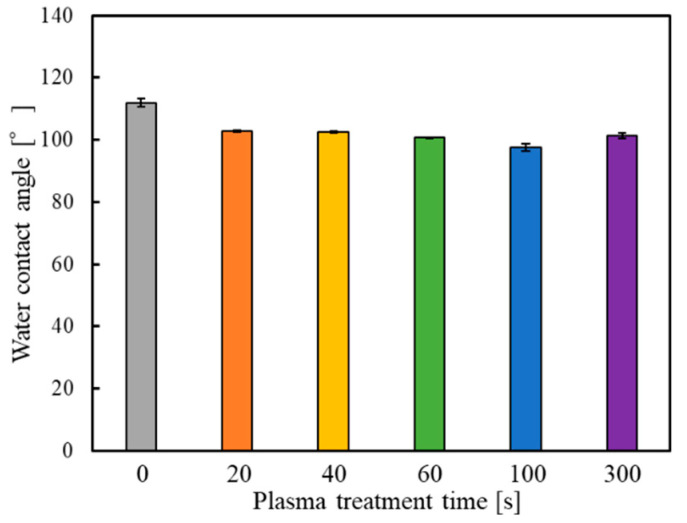
The WCAs of the GC–PTFE samples before and after Ar + H_2_O plasma treatment.

It was reported that the WCA decreased as a result of plasma treatment of the PTFE surface [40]. Figure 1 shows the WCAs of the surface of the as-received GC–PTFE and the GC–PTFE treated with Ar + H_2_O plasma for 20, 40, 60, 100, and 300 s. The WCAs of the GC–PTFE Ar + H_2_O plasma-treated for 0 (as-received), 20, 40, 60, 100, and 300 s were 111.9, 102.7, 102.5, 100.8, 97.6, and 101.3°, respectively. The WCA was decreased by Ar + H_2_O plasma treatment. However, the WCA values were constant regardless of the plasma treatment time. This is because the formation of functional groups generated at the GC–PTFE surface was constant regardless of the plasma treatment time.

### 3.2. Assessment of the Chemical-Bonding State of GC–PTFE Using XPS

**Figure 2:**
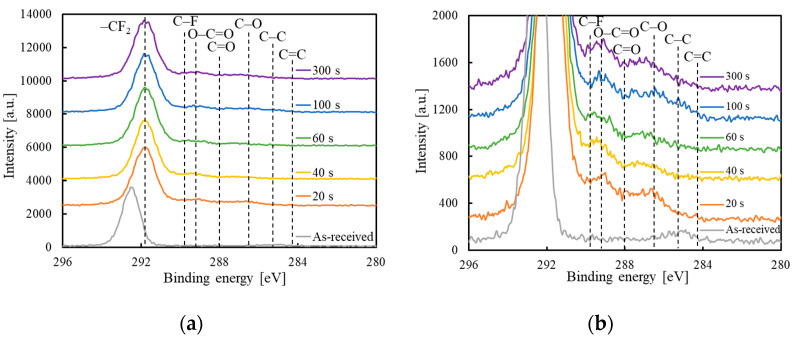
C1s-XPS spectra of the GC–PTFE samples before and after Ar + H_2_O plasma treatment: (**a**) the entire C1s-XPS spectra; (**b**) the enlarged view of C1s-XPS spectra showing the formation of functional groups.

Figure 2 shows the XPS spectra of the surface of the as-received GC–PTFE and the GC–PTFE treated with Ar + H_2_O plasma for 20, 40, 60, 100, and 300 s. Figure 2a indicates that, in the case of the as-received GC–PTFE surface, the peak at 292.5 eV, which is attributed to –CF_2_–, was observed. Figure 2b indicates that, in the case of the Ar + H_2_O plasma-treated GC–PTFE surface, the peaks at 289.2, 288.0, and 286.5 eV, which correspond to O–C=O, C=O, and –C–O–, respectively, were observed regardless of the plasma treatment time. Figure S1 (in the supplementary material) and Table 2 shows the XPS deconvolution of the GC–PTFE surface and the deconvolution ratio before and after Ar + H_2_O plasma treatment. For the as-received GC–PTFE, the ratio of CF_2_ was 96.7%. However, it decreased to less than 88% after Ar + H_2_O plasma treatment. Similarly, the formation of functional groups was constant regardless of the plasma treatment time. Thus, it can be concluded that regardless of the plasma treatment time, the F atom of the GC–PTFE surface desorbs and the oxygen-containing functional groups are generated at the GC–PTFE surface by plasma treatment. It was reported that the formation of polar groups, such as oxygen-containing functional groups, on the polymer surface decreased the WCA [26,41,42]. The fact that the formation of functional groups was constant regardless of the plasma treatment time corresponds with the fact that the WCA was constant regardless of the plasma treatment time. The Si2p-XPS spectra are illustrated in Figure S2 from the supplementary material. The peak was indexed to SiO_2_ at 103 eV [43] derived from glass cloth inside the GC–PTFE. However, the peak that corresponds to SiO_2_ was not observed at the as-received or plasma-treated GC–PTFE surface. This confirms that the glass cloth inside the GC–PTFE was not exposed to the surface of the plasma-treated GC–PTFE.

Moreover, survey XPS spectra were obtained to confirm the effect of cleaning the SUS304 foils using PJ treatment. Figure S2 and Table S1 (in the Supplementary Material) show the survey XPS spectra and elemental ratios of the SUS304 foils before and after N_2_ + Air PJ treatment, respectively [44,45,46,47]. The survey XPS spectra indicate that the peaks at 285 and 532 eV, which are attributed to carbon and oxygen, respectively, were observed for the as-received SUS304 foils. However, the intensity of the C peak was lower in the case of the PJ-treated SUS304 foils. The cleaning effect was confirmed by decreasing the intensity of C1s and increasing the intensity of O1s. Similarly, the elemental ratio of C decreased to less than half, and the ratios of Fe and O increased after the PJ treatment. This can be attributed to the removal of the carbon-containing contaminants and the exposure of the oxide layer on the SUS304 foil surface as a result of PJ treatment.

### 3.3. Surface Roughness Measurement of GC–PTFE Using CLSM

**Figure 3:**
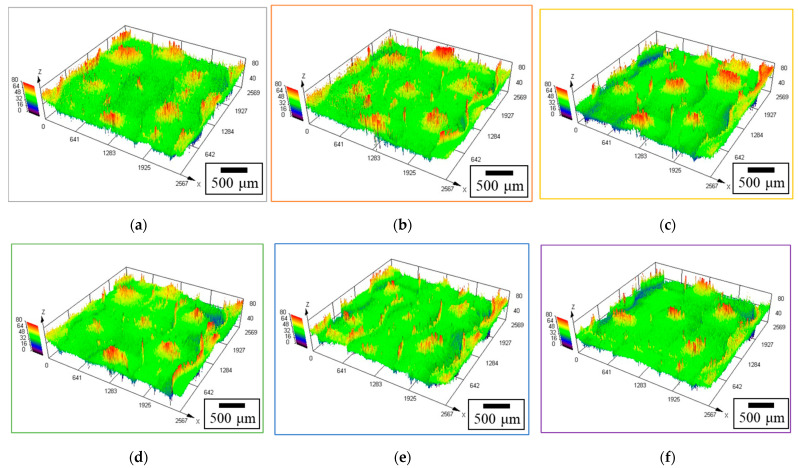
CLSM images of the GC–PTFE surface (**a**) before (as-received sample) and after Ar + H_2_O plasma treatment for (**b**) 20 s, (**c**) 40 s, (**d**) 60 s, (**e**) 100 s, and (**f**) 300 s.

The surface roughness of the GC–PTFE samples before and after Ar + H_2_O plasma treatment was investigated using CLSM. Figure 3 shows CLSM images of the as-received GC–PTFE and GC–PTFE surfaces treated with Ar + H_2_O plasma for 20–300 s. Table 3 shows the root mean square (Sq) and arithmetic mean estimation (Sa) of the surface roughness, respectively. The surface roughness of the GC–PTFE treated with Ar + H_2_O plasma for 20–100 s was higher than that of the as-received GC–PTFE. However, for the GC–PTFE treated for 300 s, the surface roughness was lower than that of the as-received GC–PTFE. This is because the amorphous part was etched on a priority basis [48] and only the amorphous part was etched when the plasma treatment time was short (20–100 s). On the other hand, when the plasma treatment time was long (300 s), both the amorphous part and the crystal part were etched. Therefore, the surface roughness increased when the plasma treatment time was short, but it decreased when the plasma treatment time was long.

### 3.4. Surface Morphology of GC–PTFE

**Figure 4:**
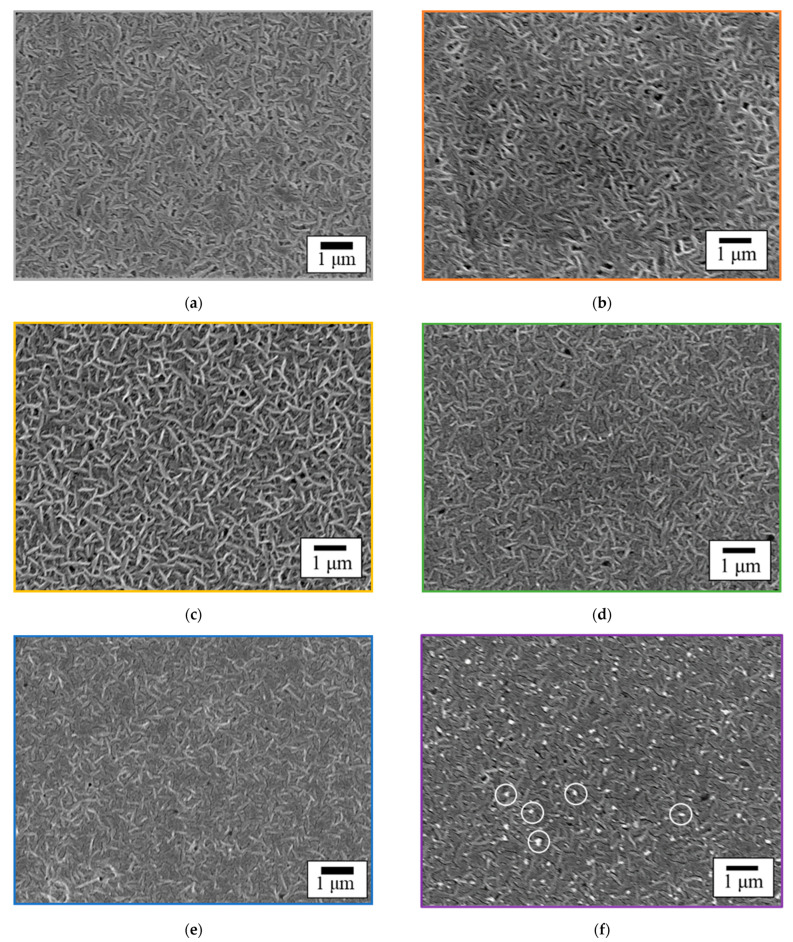
SEM images of the GC–PTFE surface (**a**) before (as-received sample) and after Ar + H_2_O plasma treatment for (**b**) 20 s, (**c**) 40 s, (**d**) 60 s, (**e**) 100 s, and (**f**) 300 s.

Figure 4 shows the SEM images of the GC–PTFE samples before and after Ar + H_2_O plasma treatment. The fibrous shape was observed on the surface of the as-received and Ar + H_2_O plasma-treated GC–PTFE. In the case of the GC–PTFE treated with Ar + H_2_O plasma for 20, 40, and 60 s, the fibrous shape on the plasma-treated PTFE was observed more clearly than on the as-received GC–PTFE. This is because the amorphous part was selectively etched by the Ar + H_2_O plasma treatment, while the fibrous shape of the crystalline part was not etched. The density of the fibrous shape decreased when the plasma treatment time was increased to 100 s. This can be attributed to the complete etching of the amorphous part by the Ar + H_2_O plasma treatment in the first 60 s, which was followed by the etching of the crystalline part. In the case of the samples treated for 300 s, several white round shapes enclosed in a white circle (diameters = ca. 0.2 μm) were observed. This can be attributed to the etching of the fibrous part, which revealed the cross-sections of the fibers.

### 3.5. Adhesion Strength Estimated by a 90° Peel Test (Plasma-Treatment Time Dependence)

**Figure 5:**
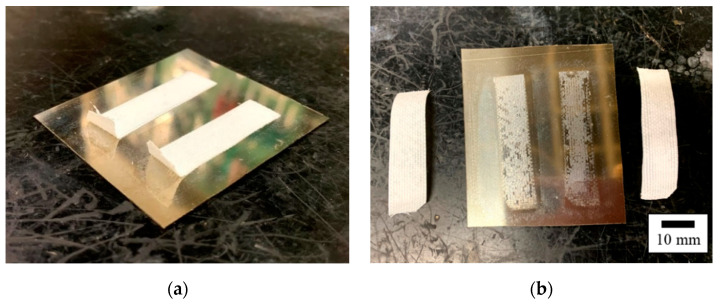
Photographs of a representative GC–PTFE/SUS304 sample (**a**) before and (**b**) after the 90° peel test.

Figure 5 shows photographs of a representative GC–PTFE/SUS304 sample before and after the peel test. In all plasma-treated GC–PTFE samples, after the peel test, the white material was found to be attached to the SUS304 foil surface (Figure 5b). This white material was the partial PTFE layer, which was originally on the GC–PTFE surface. This phenomenon reflects the dominance of the cohesion failure of the GC–PTFE.

**Figure 6:**
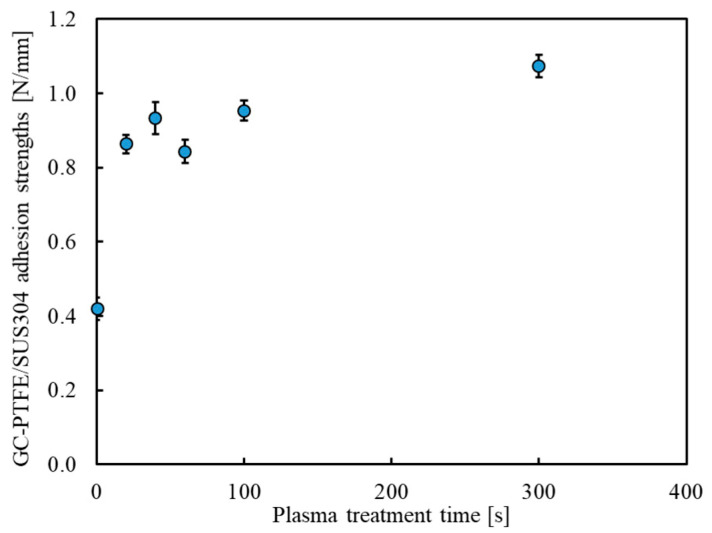
The adhesion strength between Ar + H_2_O plasma-treated GC–PTFE and SUS304 (the adhesion property of the GC–PTFE depended on the Ar + H_2_O plasma treatment time).

Figure 6 shows the adhesion strength of the GC–PTFE/SUS304 samples treated with Ar + H_2_O plasma for different times. The adhesion strengths of the GC–PTFE plasma-treated for 0 (as-received), 20, 40, 60, 100, and 300 s were 0.42, 0.86, 0.93, 0.84, 0.95, and 1.07 N/mm, respectively. A very high adhesion strength (0.8 N/mm) was achieved by Ar + H_2_O plasma treatment for only 20 s using a GC–PTFE sheet with no WBL. Furthermore, the adhesion strength was approximately 0.9 N/mm when the plasma treatment time was 0–100 s. However, an adhesion strength of 1.0 N/mm or higher was achieved with a plasma treatment time of 300 s, with the desired value being achieved. This can be attributed to the decrease in the surface roughness and the increase in the contact area of the SUS304 foils during thermal compression as a result of the plasma treatment for 300 s.

### 3.6. Comparison of the Adhesion Properties of GC–PTFE and Pure PTFE

**Figure 7:**
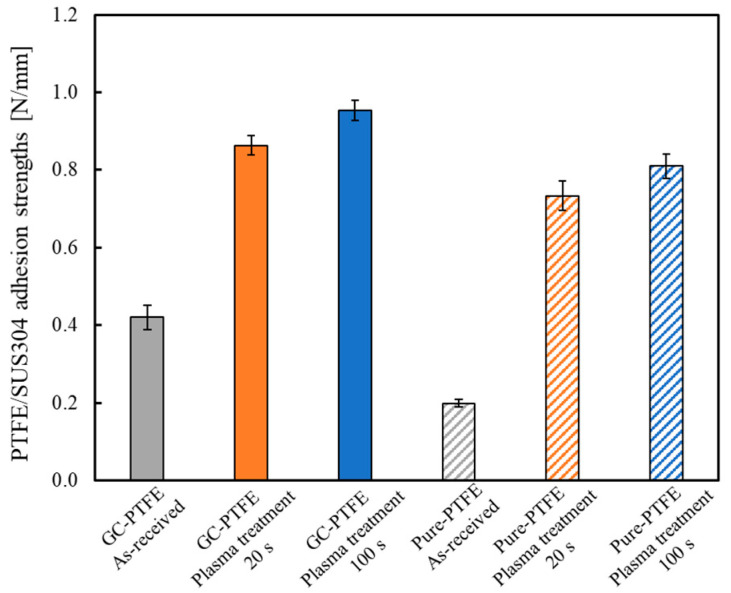
Comparison of the adhesion strengths of GC–PTFE/SUS304 and pure PTFE/SUS304 (which was dependent on the absence or presence of a WBL).

Figure 7 shows the adhesion strengths of the GC–PTFE/SUS304 and pure PTFE/SUS304 samples. The adhesion strengths of the GC–PTFE Ar + H_2_O plasma-treated for 0 (as-received), 20, and 100 s were 0.42, 0.86, and 0.84 N/mm, respectively. The adhesion strengths of the pure PTFE Ar + H_2_O plasma-treated for 0 (as-received), 20, and 100 s were 0.20, 0.73, and 0.81 N/mm, respectively. Thus, the adhesion strengths of the GC–PTFE/SUS304 were higher than those of the pure PTFE/SUS304 for both the as-received and plasma-treated PTFE samples for 20 s. This is because GC–PTFE has no WBL, and hence, no time was required to remove it, while the pure PTFE contains a WBL, and the time required to remove it was detrimental to the adhesion strength. However, when PTFE was Ar + H_2_O plasma-treated for 100 s, there were few differences in adhesion strengths between GC–PTFE and pure PTFE. This is because the WBL on the pure PTFE surface was removed when the Ar + H_2_O was plasma-treated for 100 s.

### 3.7. Comparison of the Wettabilities of GC–PTFE and Pure PTFE Using WCA Measurement

**Figure 8:**
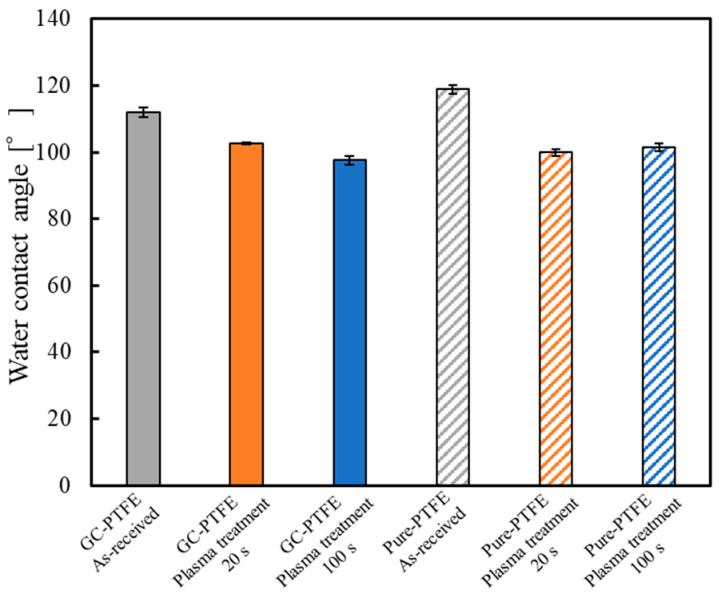
WCA of the GC–PTFE and pure PTFE samples before and after Ar + H_2_O plasma treatment.

Figure 8 shows the WCAs of the surface of the GC–PTFE and pure PTFE Ar + H_2_O plasma-treated for 0 (as-received), 20, and 100 s. The WCAs of the GC–PTFE surface Ar + H_2_O plasma-treated for 0 (as-received), 20, and 100 s were 111.9, 102.7, and 97.6°, respectively. Furthermore, the WCAs of the pure PTFE surface Ar + H_2_O plasma-treated for 0 (as-received), 20, and 100 s were 118.9, 99.9, and 101.4°, respectively. The WCA was decreased by Ar + H_2_O plasma treatment for both the GC–PTFE and pure PTFE. However, the WCA values were constant regardless of the plasma treatment time. This is because the formation of functional groups generated at the GC–PTFE and pure PTFE surface was constant regardless of the plasma treatment time.

### 3.8. Comparison of the Chemical-Bonding States of GC–PTFE and Pure PTFE

**Figure 9:**
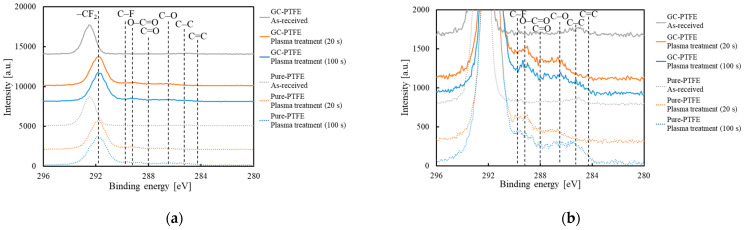
C1s-XPS spectra of the GC–PTFE and pure PTFE samples before and after Ar + H_2_O plasma treatment for 20 s and 100 s: (**a**) the entire C1s-XPS spectra and (**b**) the enlarged view of C1s-XPS spectra showing the formation of functional groups.

The chemical-bonding states of the GC–PTFE and pure PTFE surfaces were compared using XPS. Figure 9 shows the C1s-XPS spectra of the GC–PTFE and the pure PTFE surfaces before and after Ar + H_2_O plasma treatment for 20 s and 100 s. For both the as-received GC–PTFE and pure PTFE surfaces, the peak corresponding to –CF_2_– (292.5 eV) was observed. For both the plasma-treated GC–PTFE and pure PTFE surfaces, peaks corresponding to O–C=O (289.2 eV), C=O (288.0 eV), and –C–O– (286.5 eV) were observed. The shape of the C1s-XPS spectra of the Ar + H_2_O plasma-treated GC–PTFE was almost the same as that of the Ar + H_2_O plasma-treated pure PTFE, which indicates that the chemical bonding states of the GC–PTFE and pure PTFE surfaces were similar. Figure S4 and Table 4 show the XPS deconvolution of the PTFE surface and the deconvolution ratio before and after Ar + H_2_O plasma treatment. For the as-received pure PTFE, the ratio of CF_2_ was 98.3%. However, it decreased to less than 90% and the oxygen-containing functional groups were generated by Ar + H_2_O plasma treatment. For both the pure PTFE and GC–PTFE surfaces, the formation of functional groups was constant regardless of the plasma treatment time. Therefore, the chemical bonding state was not the factor causing the difference in the adhesion strengths between GC–PTFE/SUS304 and pure PTFE/SUS304.

### 3.9. Comparison of the Surface Morphology of GC–PTFE and Pure PTFE

**Figure 10:**
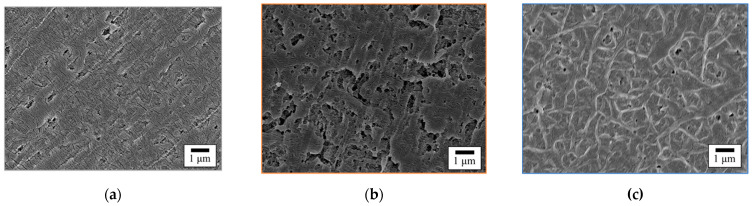
The SEM images of the pure PTFE samples (**a**) before (as-received), (**b**) after 20 s of the Ar + H_2_O plasma treatment, and (**c**) after 100 s of the Ar + H_2_O plasma treatment.

Figure 10 shows the SEM images of the pure PTFE samples before and after Ar + H_2_O plasma treatment for 20 s and 100 s. In contrast to the SEM images of the GC–PTFE with no WBL (Figure 4a,b), voids of several μm in size can be seen on the surface of as-received pure PTFE. These voids were introduced by the cutting process and are evidence of the existence of a WBL. The damage caused by the cutting process was observed on the as-received pure PTFE surface (Figure 10a). The voids were observed on the surface of the pure PTFE treated with Ar + H_2_O plasma for 20 s. The damage caused by the cutting process was also observed on the surface of the pure PTFE treated with Ar + H_2_O plasma for 20 s, which confirms that the WBL remained after the treatment (Figure 10b). The comparison of the SEM images of the GC–PTFE and pure PTFE surfaces revealed that the as-received and pure PTFE Ar + H_2_O plasma-treated for 20 s had a WBL, while the as-received and GC–PTFE Ar + H_2_O plasma-treated for 20 s had no WBL. This is why the adhesion strength of the pure PTFE/SUS304 was lower than that of the GC–PTFE/SUS304 when the plasma treatment time was short. However, the voids were observed on the pure PTFE Ar + H_2_O plasma-treated for 100 s (Figure 10c), showing that the WBL was removed when the Ar + H_2_O was plasma treatment for 100 s. Therefore, there were few differences in the adhesion strength between the GC–PTFE and pure PTFE when the plasma treatment time was 100 s.

## 4. Conclusions

In this study, the effect of Ar + H_2_O plasma treatment on the adhesion properties of GC–PTFE with no WBL was studied. The WCA of the GC–PTFE decreased after Ar + H_2_O plasma treatment. The generation of oxygen-containing functional groups on the surface of the Ar + H_2_O plasma-treated GC–PTFE was confirmed by C1s-XPS spectra. The Si2p-XPS spectra showed that no exposed glass cloth was observed on the surface of the Ar + H_2_O plasma-treated GC–PTFE. The CLSM and SEM results indicated that the surface roughness increased by etching only the amorphous parts when the plasma-treatment time was short (< 60 s), and decreased when the plasma-treatment time was long (> 100 s) because both the amorphous and crystalline parts were etched. A strong adhesion strength of 0.8 N/mm was obtained between GC–PTFE treated with Ar + H_2_O plasma for 20 s and SUS304 foils. When both the GC–PTFE and pure PTFE samples were plasma-treated under the same conditions, the adhesion strength of GC–PTFE/SUS304 was higher than that of pure PTFE/SUS304 when the plasma treatment time was short. There was little difference between the chemical bonding states of Ar + H_2_O plasma-treated GC–PTFE and pure PTFE. However, there was a big difference in the surface morphologies of Ar + H_2_O plasma-treated GC–PTFE and pure PTFE. Although some cutting scratches remained on the Ar + H_2_O plasma-treated pure PTFE, no cutting scratches were observed on the Ar + H_2_O plasma-treated GC–PTFE when the plasma treatment time was 20 s. Thus, the adhesion strength of pure PTFE/SUS304 was lower than that of GC–PTFE/SUS304 because of the remaining WBL on the plasma-treated pure PTFE surface when the plasma treatment time was 20 s. The WBL does not exist on the GC–PTFE surface, so its removal is not necessary. However, in order to adhere to other materials, the oxygen-containing functional groups need to be introduced onto the GC–PTFE surface by plasma treatment, because it does not exist on the GC–PTFE surface. For the pure PTFE, both the removal of the WBL and the formation of oxygen-containing functional groups are necessary when using plasma treatment because the WBL exists on the pure PTFE surface. When the plasma treatment time is short, the oxygen-containing functional groups can be generated, but the WBL is not removed. Therefore, pure PTFE requires a longer plasma treatment time than GC–PTFE. Thus, when pure PTFE with a WBL is replaced with GC–PTFE, which has no WBL, a shorter plasma treatment time is required to achieve a direct strong adhesion of >0.8 N/mm to SUS304 foil. A method for manufacturing PTFE with no WBL, the use of other fluoropolymers with no WBL, and a high-speed method for removing or recovering a WBL should be considered in future studies.

## Figures and Tables

**Table 1:** The surface-modification conditions and adhesion properties for different cases of PTFE.

Material	Adherend	Surface Modification Treatment	Adhesion Strength	Adhesive	References
Pure PTFE	Cu	Ar^+^ ion irradiation	No data	No	[9]
Pure PTFE	Glass-cloth	Nitrogen ion irradiation	0.02 to 0.9 N/mm	An epoxy adhesive	[10,11]
Pure PTFE	PMMA	UV irradiation using 1,2-diaminoethame	0.03 to 10 MPa	An epoxy adhesive	[13]
Pure PTFE	Plexiglass	UV irradiation using triethylene tetramine	0.03 to 9 MPa	An epoxy adhesive	[14]
Pure PTFE	CFRP	EB irradiation	0.1 N/mm	No	[22]
Pure PTFE	PDMS	EB irradiation	0.11 N/mm	No	[23]
Pure PTFE	Fe	Ar + H_2_O plasma treatment	0.3 to 5.8 MPa	An epoxy adhesive	[29]
Pure PTFE	NBR	NH_3_ plasma treatment	8.1 N/mm	A phenolic adhesive	[30]
Pure PTFE	Al	Ar + acrylic acid plasma treatment	1.4 N/mm	An epoxy adhesive	[31]
Pure PTFE	Stainless steel	Heat-assisted Ar plasma treatment	0.00 to 1.17 N/mm	An epoxy adhesive	[32]
Pure PTFE	Cu	Plasma polymerization using GMA	0.03 to 0.48 N/mm	No	[33]
Pure PTFE	Cu	Plasma polymerization using VIDz and GMA	1.9 N/mm	No	[34]
Pure PTFE	IIR	Heat-assisted He plasma treatment	0.1 to > 2.0 N/mm	No	[35]
Pure PTFE	Ag	Open-air type Ar + H_2_O plasma treatment	0.04 to > 1.0 N/mm	No	[36]
GC–PTFE	Stainless steel	Open-air type Ar + H_2_O plasma treatment	0.42 to > 1.07 N/mm	No	This study

**Table 2:** Deconvolution ratio of C1s-XPS spectra of the GC–PTFE surface before and after Ar + H_2_O plasma treatment with different treatment times.

	CF_2_ [%]	C–F [%]	O–C=O [%]	C=O [%]	C–O [%]	C–C [%]	C=C [%]
0 s	96.7	0.0	0.0	0.0	0.0	3.3	0.0
20 s	81.6	4.5	3.0	5.2	4.5	1.2	0.0
40 s	87.2	3.3	4.0	2.5	3.0	0.2	0.0
60 s	88.0	5.1	2.5	2.5	1.4	0.5	0.0
100 s	80.3	4.2	3.8	4.5	4.5	2.3	0.5
300 s	81.3	4.1	4.7	4.5	4.6	0.8	0.2

**Table 3:** Surface roughness of the GC–PTFE surface before and after the Ar + H_2_O plasma treatment. The measurements were repeated three times and the average was defined as the surface roughness.

Treatment Time [s]	0	20	40	60	100	300
Sq [μm]	7.92 ± 0.25	8.82 ± 0.24	8.49 ± 0.19	8.79 ± 0.17	8.12 ± 0.13	7.29 ± 0.23
Sa [μm]	5.85 ± 0.27	6.08 ± 0.24	6.31 ± 0.15	6.60 ± 0.14	6.06 ± 0.13	5.30 ± 0.16

**Table 4:** Comparison of the deconvolution ratios of C1s-XPS spectra of the GC–PTFE and pure PTFE surface before and after Ar + H_2_O plasma treatment.

Sample		CF_2_ [%]	C–F [%]	O–C=O [%]	C=O [%]	C–O [%]	C–C [%]	C=C [%]
GC–PTFE	0 s	96.7	0.0	0.0	0.0	0.0	3.3	0.0
	20 s	81.6	4.5	3.0	5.2	4.5	1.2	0.0
	100 s	80.3	4.2	3.8	4.5	4.5	2.3	0.5
Pure PTFE	0 s	98.3	0.0	0.0	0.0	0.0	1.7	0.0
	20 s	89.1	1.4	4.3	2.8	2.5	0.0	0.0
	100 s	81.7	1.9	4.9	2.9	4.3	4.4	0.0

## Data Availability

The data presented in this study are available in article.

## Supplementary Materials

The following supporting information can be downloaded at https://www.mdpi.com/article/10.3390/polym14030394/s1, Figure S1: Deconvolution of C1s-XPS spectra of the GC–PTFE surface (a) before (as-received sample) and after Ar + H_2_O plasma treatment for (b) 20 s, (c) 40 s, (d) 60 s, (e) 100 s, and (f) 300 s, Figure S2: Si2p-XPS spectra of the GC–PTFE samples before and after Ar + H_2_O plasma treatment, Figure S3: Survey XPS spectra of the SUS304 foils before and after N_2_ + Air PJ treatment to confirm the effect of cleaning the SUS304 foils using PJ treatment, Figure S4: Deconvolution of C1s-XPS spectra of the pure PTFE surface (a) before (as-received sample) and after Ar + H_2_O plasma treatment for (b) 20 s, (c) 100 s, Table S1: Atomic ratios of the SUS304 foils before and after N_2_ + Air PJ treatment.
