# Supplementary material for: Adhesive-Free Adhesion between Plasma-Treated Glass-Cloth-Containing Polytetrafluoroethylene (GC–PTFE) and Stainless Steel: Comparison between GC–PTFE and Pure PTFE
Source: Polymers (Basel). 2022 Jan 20;14(3):394. doi: 10.3390/polym14030394 (PMC8838903; doi:10.3390/polym14030394)
Supplement: Supplementary file 1 [file polymers-14-00394-s001.zip › polymers-1526207-supplementary.pdf]

Supplementary Information

Adhesive-free Adhesion between Plasma-treated Glass-cloth-containing Polytetrafluoroethylene (GC-PTFE) and Stainless Steel: Comparison between GC-PTFE and Pure-PTFE

Misa Nishino [1], Yuki Okazaki [1], Yosuke Seto [1], Tsuyoshi Uehara[2], Katsuyoshi Endo [1], Kazuya Yamamura [1], and Yuji Ohkubo [1,*]

[1]Graduate School of Engineering, Osaka University,2-1 Yamadaoka, Suita, Osaka, 565-0871, Japan;

[2]SEKISUI CHEMICAL Co., Ltd., 2-2 Kamichoshi-cho, Kamitoba, Minami-ku, Kyoto, 601-8105, Japan;

Contents

●XPS deconvolutions of GC-PTFE before and after Ar + H_2O plasma treatment..... Figure S1

●Si2p-XPS spectra of GC-PTFE before and after Ar + H_2O plasma treatment........... Figure S2

●Survey XPS spectra of the SUS304 foils before and after N_2 + Air PJ treatment...... Figure S3

●Atomic ratios of the SUS304 foils before and after N_2 + Air PJ treatment................. Table S1

●XPS deconvolutions of pure-PTFE before and after Ar + H_2O plasma treatment....Figure S4

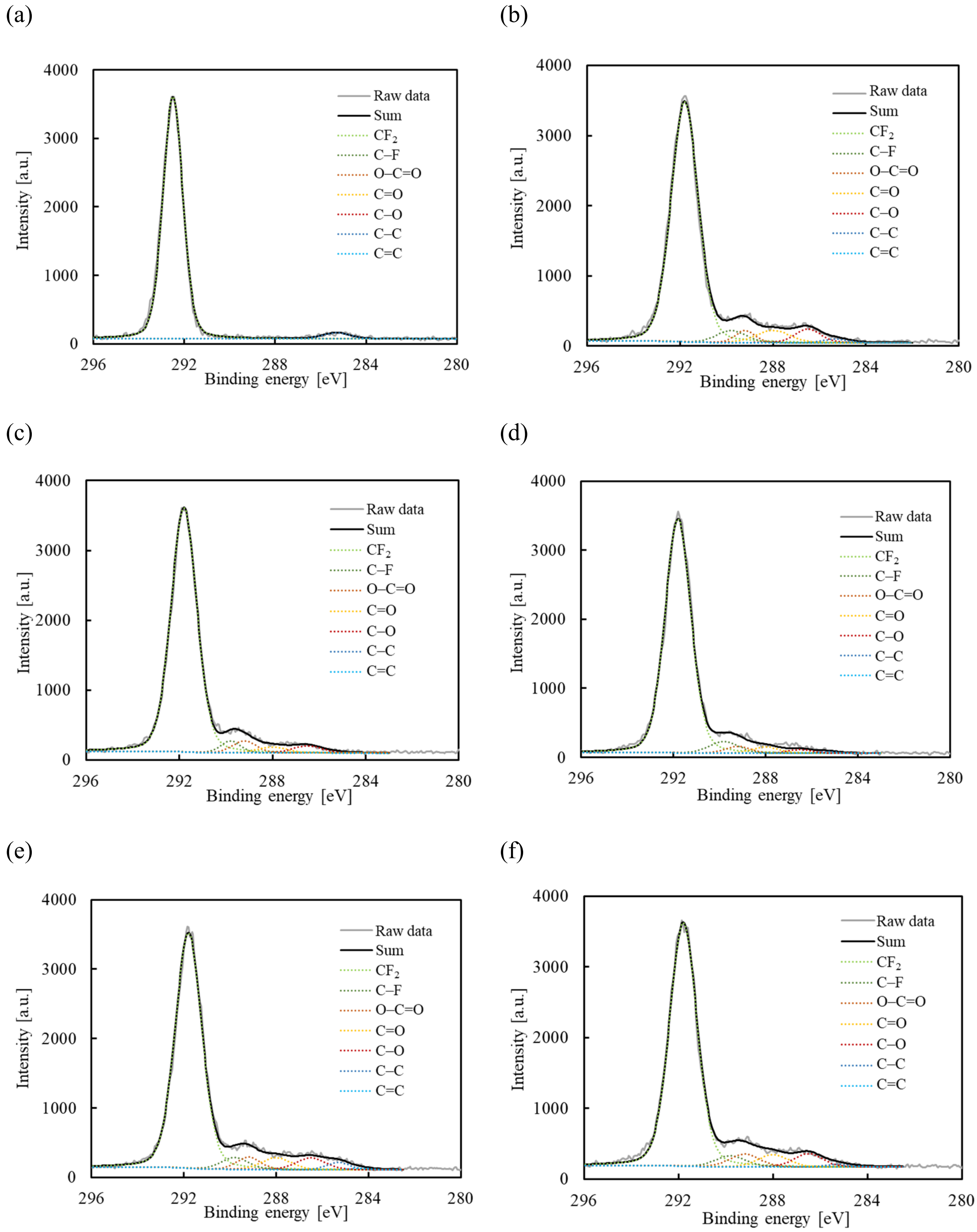

Figure S1. Deconvolution of C1s-XPS spectra of the GC-PTFE surface (**a**) before (as-received sample) and after Ar + H_2O plasma treatment for (**b**) 20 s, (**c**) 40 s, (**d**) 60 s, (**e**) 100 s, and (**f**) 300 s.

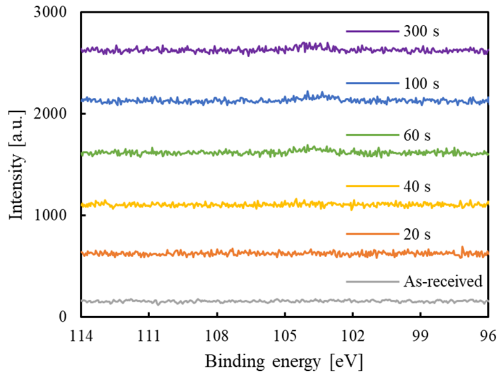

Figure S2. Si2p-XPS spectra of the GC-PTFE samples before and after Ar + H_2O plasma treatment.

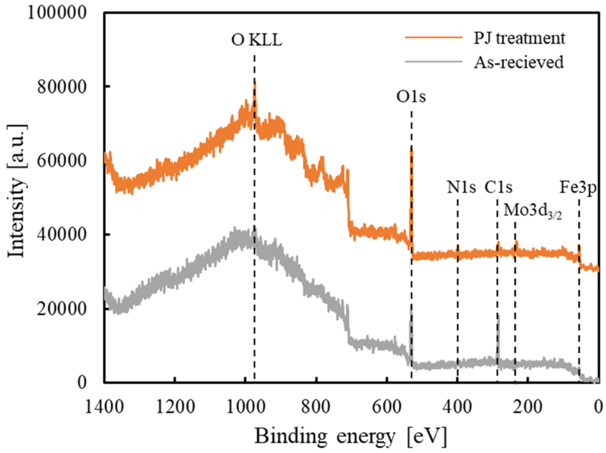

Figure S3. Survey XPS spectra of the SUS304 foils before and after N_2 + Air PJ treatment to confirm the effect of cleaning the SUS304 foils using PJ treatment.

Table S1. Atomic ratios of the SUS304 foils before and after N_2 + Air PJ treatment.

	Fe [at%]	C [at%]	O [at%]	N [at%]
As-received	2.4 ± 1.1	59.1 ± 5.3	36.5 ± 3.8	2.0 ± 0.9
PJ treatment	9.3 ± 0.7	19.6 ± 2.9	63.7 ± 3.2	7.4 ± 0.7

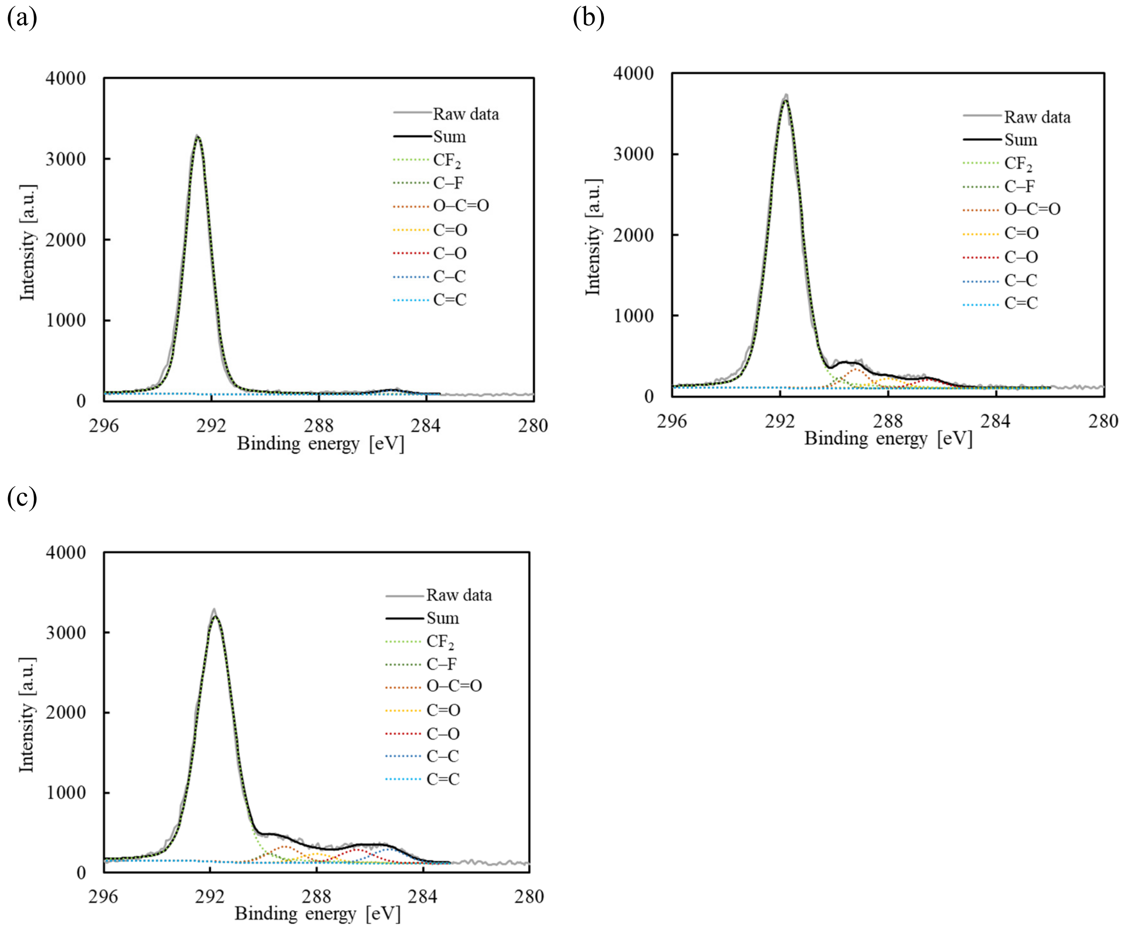

Figure S4. Deconvolution of C1s-XPS spectra of the pure-PTFE surface (**a**) before (as-received sample) and after Ar + H_2O plasma treatment for (**b**) 20 s, (**c**) 100 s
